# Effect of ciprofol on the incidence of hypotension during induction of general anesthesia in elderly patients undergoing total hip arthroplasty: single-center retrospective study

**DOI:** 10.3389/fmed.2026.1764580

**Published:** 2026-02-09

**Authors:** Jiaxi Zhu, Yun Chen, Ying Cao, Yicai Gan, Xingan Zhang, Bo Xu

**Affiliations:** 1 Department of Anesthesiology, General Hospital of Southern Theater Command, Guangzhou, Guangdong, China; 2 The First School of Clinical Medicine, Southern Medical University, Guangzhou, Guangdong, China

**Keywords:** ciprofol, elderly, general anesthesia, post-induction hypotension, propofol

## Abstract

**Objective:**

Post-induction hypotension (PIH) represents a frequent perioperative complication among elderly patients, and its prevention is critical for reducing organ damage and postoperative adverse outcomes. This study aims to evaluate the impact of ciprofol on the incidence of PIH when used for general anesthesia induction in elderly patients.

**Methods:**

A retrospective review was carried out on the clinical records of elderly patients who underwent elective total hip replacement surgery at our hospital between August 2024 and July 2025. Patients were stratified into the ciprofol group and the propofol group based on the anesthetic sedative agents administered. The primary outcome indicator was PIH, characterized as a mean arterial pressure (MAP) ≤65 mmHg or a reduction greater than 30% relative to baseline values within 20 min post-induction. Subgroup analyses included considerations of preoperative hypertension, the Clinical Frailty Scale (CFS), the age-adjusted Charlson Comorbidity Index (aCCI), and patient age.

**Results:**

Baseline clinical features were comparable across groups. The incidence of PIH was markedly lower in the ciprofol group than in the propofol group (36.4% vs. 65.5%; absolute risk reduction = 29.1, 95% confidence interval: 11.2–47.0%, *p* = 0.002). Subgroup analyses demonstrated that the advantages of ciprofol remained consistent across all pre-specified subgroups, with prominent differences noted in patients aged under 90 years, those with an aCCI of ≤4, a CFS score of ≤4, and baseline blood pressure (BP) of <140/90 mmHg.

**Conclusion:**

Ciprofol reduces PIH risk, improves haemodynamic stability, and decreases vasoactive agent use and injection pain compared with propofol in elderly patients, independent of baseline characteristics, indicating suitability for anesthetic management in this population.

## 1 Introduction

Post-induction hypotension (PIH) remains the most prevalent form of perioperative hypotension, with established associations to increased risks of organ hypoperfusion (1). This condition is linked to adverse postoperative outcomes, including renal dysfunction, myocardial injury, postoperative delirium, ischemic stroke, and elevated 30 day mortality (2, 3). Notably, patient-specific factors (e.g., anesthetic agent responsiveness) rather than surgical variables (e.g., procedure duration, invasiveness, or intraoperative blood loss) predominantly influence PIH occurrence (3). Elderly patients exhibit a disproportionately higher PIH incidence due to reduced physiological reserve, comorbidity burden, and heightened sensitivity to anesthetic agents, underscoring the clinical imperative to optimize PIH preventive strategies—particularly through rational induction agent selection (4, 5).

Propofol, a widely used intravenous anesthetic, is valued for its rapid onset, short half-life, and high-quality recovery (6). However, its clinical utility is constrained by notable limitations: injection pain (7), respiratory depression, and cardiovascular adverse effects (hypotension, bradycardia, and arrhythmia) (8). Circulatory depression stems from peripheral vasodilation and myocardial depression, effects that are accentuated in elderly and frail populations (9, 10). Studies confirm that propofol induction in older adults increases hypotension risk, which correlates with poor outcomes (11).

Ciprofol, a novel intravenous anesthetic with cyclopropyl group modification, exhibits enhanced stereoselectivity and 4-5-fold higher *γ*-aminobutyric acid type A (GABAA) receptor affinity compared to propofol, requiring only 1/5 of the equivalent concentration for comparable receptor-mediated effects (12). Phase III trials demonstrate that ciprofol’s individualized dosage is approximately 1/4 to 1/5 that of propofol (13). Research indicates ciprofol retains propofol’s pharmacokinetic advantages while offering potential benefits: low injection pain incidence, high potency, wide therapeutic window, and milder cardiorespiratory depression (14, 15). Preliminary evidence supports its safety and efficacy in geriatric general anesthesia (16, 17). Despite these attributes, high-quality evidence specific to elderly populations remains limited, necessitating further validation.

In this study, by retrospectively analyzing the clinical data of elderly patients undergoing total hip arthroplasty (THA) and comparing the incidence of hypotension during anesthesia induction between ciprofol and propofol, we aim to evaluate the safety of ciprofol in general anesthesia induction for elderly patients undergoing orthopedic surgery, thereby providing clinical evidence for optimizing anesthesia induction protocols in elderly patients.

## 2 Materials and methods

### 2.1 Study design and population

This study constitutes a single-center, retrospective observational investigation. The study protocol underwent review and approval by the Ethics Committee of Southern Theater Command General Hospital (No. NZLLKZ2024150). This study complies with the regulations concerning human trials as specified in the World Medical Association Declaration of Helsinki-Ethical Principles for Medical Research Involving Human Subjects. Due to the retrospective design of this study and the anonymization of patient information, the institutional review board waived the requirement for informed consent from subjects. This study follows the STROBE guidelines for observational studies (18). Patients included in this study were elderly individuals who underwent orthopedic surgery under general anesthesia in our hospital from August 2024 to July 2025. They were divided into the ciprofol group and the propofol group based on the sedative drugs used for anesthesia induction. Inclusion criteria: (1) Age ≥65 years; (2) Undergo elective THA under general anesthesia; (3) American Society of Anesthesiologists (ASA) physical status classification I–III; (4) Body mass index (BMI) ranging from 18 to 30 kg·m^−2^. Exclusion criteria: (1) Suffering from severe cardiovascular illnesses or serious arrhythmias; (2) Having severe functional impairment of the heart, lung, liver, or kidney; (3) Having a history of mental illnesses, long-term use of psychotropic medications (such as dementia, schizophrenia), chronic painkillers, or alcohol addiction; (4) Having taken sedatives or anesthetic in the previous 24 h; (5) Being allergic to the study drugs.

### 2.2 Perioperative management

All patients followed a standardized perioperative fluid management protocol: Prior to surgery, they abstained from solid foods for 8 h and clear liquids for 4 h. Upon arrival in the operating room, peripheral intravenous access was promptly established. Within 30 min before anesthesia induction, an intravenous infusion of compound sodium lactate solution (10 mL·kg^−1^) was administered for prophylactic fluid loading to reduce the risk of PIH related to hypovolemia. Routine non-invasive monitoring was conducted, encompassing electrocardiography (ECG), pulse oximetry (SpO₂), heart rate (HR), and Bispectral Index (BIS). Additionally, radial artery puncture and catheterization were carried out to enable continuous invasive arterial blood pressure monitoring.

Participants assigned to the ciprofol group were administered an intravenous dose of sufentanil (Yichang Humanwell Pharmaceutical, Yichang, China) at 0.3–0.5 μg·kg^−1^, after which ciprofol (Liaoning Hisoar Pharmaceutical, Liaoning, China) was slowly infused intravenously (over 60 s) at 0.4 mg·kg^−1^. For the propofol group, intravenous sufentanil (0.3–0.5 μg·kg^−1^) was administered in combination with a slow intravenous infusion of propofol (Liaoning Hisoar Pharmaceutical) at 2.0 mg·kg^−1^ over a 60 s period. Once sufficient sedation was achieved [Modified Observer’s Assessment of Alertness/Sedation (MOAA/S) score ≤1; Supplementary Table S1], cisatracurium (0.15–0.2 mg·kg^−1^) was given intravenously to assist with tracheal intubation. No other sedative drugs such as midazolam or dexmedetomidine, nor any adjuvant medications were administered during the induction.

Anesthesia maintenance was achieved via continuous intravenous infusion of ciprofol (0.8–2.4 mg·kg^−1^·h^−1^) or propofol (4–6 mg·kg^−1^·h^−1^), and both groups were administered remifentanil via target-controlled infusion (Minto model, target effect-site concentration: 5 ng·mL^−1^). Rates of infusion were adjusted in real time to keep BIS values within the 40–60. Intraoperatively, pressure-controlled ventilation was utilized to maintain end-tidal carbon dioxide (PETCO₂) at 35–45 mmHg and oxygen saturation (SpO₂) at 90% or above. During the induction and maintenance of anesthesia, the patients’ circulatory hemodynamic status was closely monitored. Hypotension was defined as an absolute MAP ≤ 65 mmHg, or a decrease of more than 30% from the baseline level. Once this criterion was met, phenylephrine 30–50 μg should be immediately administered intravenously for correction. Bradycardia was defined as a heart rate (HR) <60 beats per minute, at which point ephedrine 5–10 mg should be given intravenously. If circulatory fluctuations persisted unrelieved 1 min after the initial administration, the above drugs could be repeated to maintain hemodynamic stability.

### 2.3 Outcomes

The primary endpoint was the incidence of PIH, defined as an absolute MAP of ≤65 mmHg or a relative decrease in MAP exceeding 30% from the baseline value within 20 min after induction or between induction and the start of surgery (19, 20). Baseline MAP was defined as the average value of MAP measured within 3 min immediately prior to anesthetic induction. Arterial blood pressure readings were automatically recorded at 1 min intervals.

Secondary outcomes included: (1) Minimum HR after loss of consciousness (LoC); (2) Number of patients receiving vasopressors and administration frequency within 20 min post-induction (or before the start of surgery); (3) Time from anesthesia initiation to LoC; (4) Time to full consciousness after drug discontinuation; (5) Incidence of injection pain; (6) Changes in HR, SBP, diastolic blood pressure (DBP), MAP, and BIS at six time points: pre-induction (T0), When BIS is ≤60 (T1), 1 min after induction (T2), intubation (T3), 3 min after intubation (T4), and 5 min after intubation (T5).

### 2.4 Statistical analysis

Statistical analyses in this study were performed using IBM SPSS Statistics 26.0 (IBM Corp., Armonk, NY, United States) and GraphPad Prism 8.1 (Boston, MA, United States). The normality of continuous variables was determined via the Shapiro–Wilk test: variables conforming to a normal distribution were expressed as mean ± standard deviation (*x̅* ± *s*), and intergroup comparisons were conducted using the independent samples *t*-test; variables with a non-normal distribution were presented as median (interquartile range) [*M* (P25, P75)], and intergroup comparisons were performed using the Mann–Whitney *U* test.

Categorical data were described as number (percentage), and intergroup comparisons were carried out using the chi-square test or Fisher’s exact test as appropriate. Repeated measures analysis of variance (ANOVA), followed by *post hoc* simple effects analysis, was used to evaluate the temporal variation trends of hemodynamic indicators and the differences between different treatment groups.

Subgroup analyses employed multivariable logistic regression models stratified by: age (<90 vs. ≥90 years), sex (male vs. female), ASA class (I-II vs. III–V), age-adjusted Charlson Comorbidity Index (aCCI ≤4 vs. >4; Supplementary Table S2), Clinical Frailty Scale (CFS ≤4 vs. >4; Supplementary Table S3), and baseline ward blood pressure (<140/90 vs. ≥140/90 mmHg). The Mantel-Haenszel method was used to calculate the odds ratios (OR) and 95% confidence intervals (CI). Subgroup data were expressed as frequency (%), and the likelihood ratio test was employed to evaluate the interaction effects among various factors. Forest plots were used for intuitive visual presentation of the analysis results. Regression analyses were performed using R software version 4.2.1. A two-sided *p*-value < 0.05 was considered statistically significant.

## 3 Results

**Figure 1:**
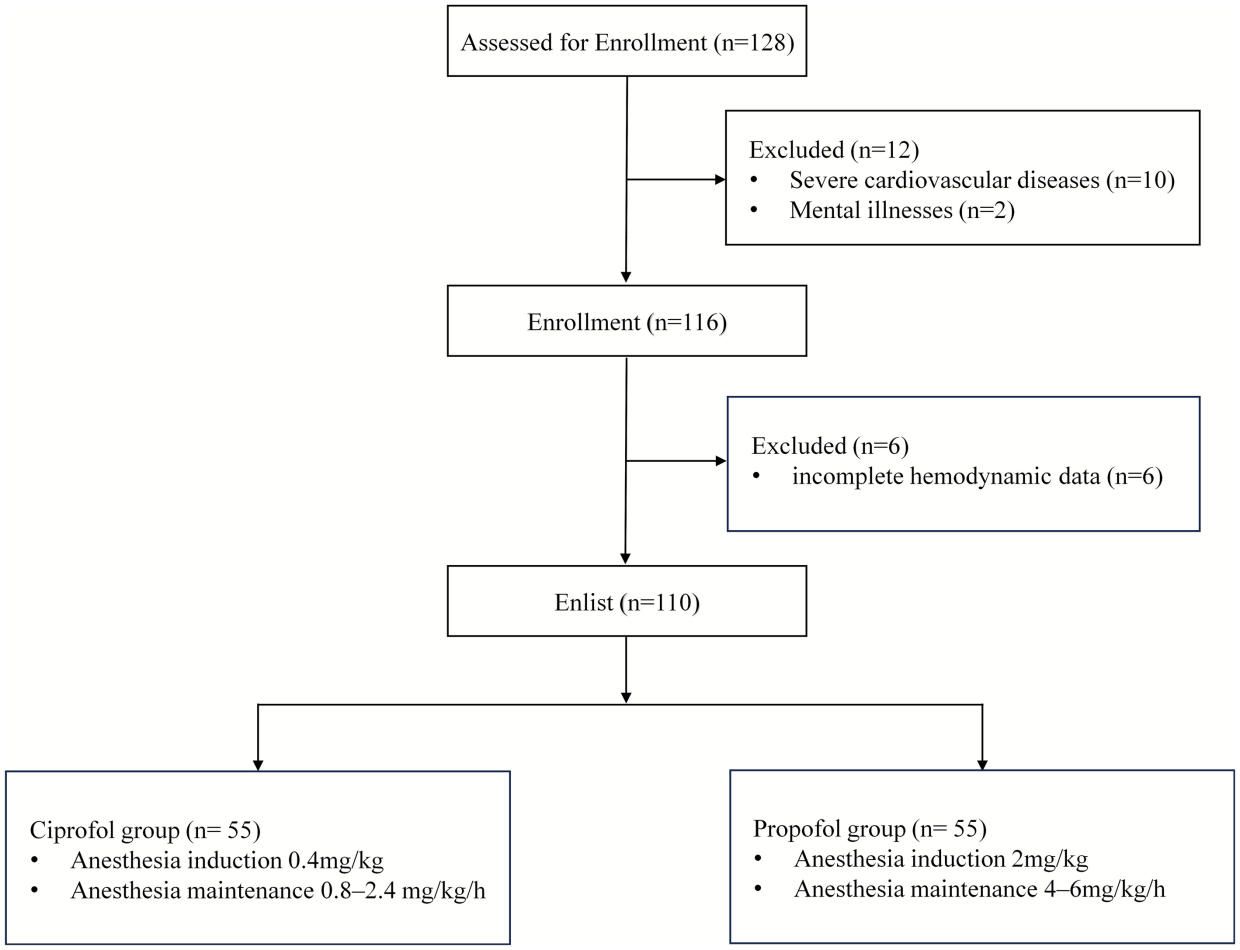
Flow chart of this study.

This retrospective investigation enrolled 128 elderly patients who underwent THA at our institution between August 2024 and July 2025, with their clinical results assessed. According to the preset exclusion criteria, a total of 18 patients were excluded, specifically including: 10 cases with severe cardiovascular diseases, 6 cases with incomplete hemodynamic data, and 2 cases with mental illnesses. Ultimately, 110 patients were enrolled in the study and included in the analysis, with 55 assigned to the ciprofol group and 55 to the propofol group (Figure 1). As presented in Table 1, the two groups showed balanced distribution in baseline demographic traits and clinical characteristics. With the exception of CFS scores, other indicators exhibited no statistically significant differences (*p* > 0.05).

**Table 1:** Subject characteristics and surgical details.

Variables	Ciprofol (*n* = 55)	Propofol (*n* = 55)	*p-*value
Age, yr	73 [69–76]	73 [70–79]	0.758
≥90 yr	3 (5.5%)	5 (9.1%)	0.716
Sex, female	33 (60%)	28 (50.9%)	0.328
Height, cm	160.1 (7.2)	162.5 (8.6)	0.115
Weight, kg	62.0 (8.5)	61.0 (10.9)	0.593
Body mass index, kg·m^−2^	24.1 (2.5)	23.1 (3.8)	0.106
Age-adjusted Charlson Comorbidity Index	4 [3–5]	4 [4-4]	0.099
CFS	3 [3-4]	3 [3-4]	0.007
ASA physical status 1-2/3-4	36/19	33/22	0.542
Medical history			
Hypertension	26 (47.3%)	32 (58.2%)	0.218
Diabetes mellitus	10 (18.2%)	13 (23.6)	0.507
Atrial fibrillation	5 (9.1%)	3 (5.5%)	0.719
Coronary artery disease	7 (12.7%)	5 (9.1%)	0.546
Respiratory disease	1 (1.8%)	4 (7.3%)	0.364
Preoperative medication			
β-blockers	18 (32.7%)	22 (40.0%)	0.552
Calcium channel blockers	15 (27.3%)	17 (30.9%)	0.834
Sleep medication use	8 (14.5%)	10 (18.2%)	0.622
Benzodiazepine	3 (5.5%)	5 (9.1%)	0.719
BP ≥140/90 mm Hg in the ward	32 (58.2%)	28 (50.9%)	0.548
Preoperative laboratory data			
Albumin, g·dl^−1^	3.97 (0.47)	3.9 (0.4)	0.402
Creatinine, mg·dl^−1^	0.8 (0.28)	0.8 (0.29)	1.000
eGFR, ml·min^−1^ 1.73·m^−2^	80.8 (23.4)	89.5 (24.3)	0.059
Hb, g·dl^−1^	12.6 (1.7)	12.2 (2.0)	0.261
Left ventricular ejection fraction (%)	63.6 (3.8)	62.3 (4.0)	0.084
Education			0.891
Illiteracy	10 (18.2%)	8 (14.5%)	
Primary	32 (58.2%)	36 (65.5%)	
Secondary	7 (12.7%)	6 (10.9%)	
University	6 (10.9%)	5 (9.1%)	
Smoking, *n* (%), yes	8 (14.5%)	6 (10.9%)	0.581
Sufentanil used during anesthetic induction (μg)	21.5 (3.2)	21.8 (3.0)	0.613
Preoperative fluid replacement volume (mL)	350 (100)	370 (100)	0.297
Time from induction to the start of surgery (min)	22.8 (5.8)	23.6 (6.2)	0.486

All data are presented as mean (SD), median [inter-quartile range], or number (%). eGFR was calculated using the modified Modification of Diet in Renal Disease (MDRD) formula: eGFR (ml/min/1.73m^2^) = 175 × (Scr)^−1.234^ × (age)^−0.179^ × (0.79 for female patients).

ASA, American Society of Anesthesiologists; BP, blood pressure; eGFR, estimated glomerular filtration rate; SD, standard deviation.

The data presented in Table 2 demonstrate a substantial reduction in the incidence of PIH in the ciprofol group when compared with the propofol group (36.4% vs. 65.5%; ARR = 29.1, 95% CI: 11.2–47.0%; *p* = 0.002). No statistically significant difference was noted in the baseline MAP between the two groups (80 ± 10 vs. 77 ± 7; SMD = 0.35, 95% CI: −0.03–0.72; *p* = 0.071). After induction, the HR in the ciprofol group was markedly higher than the propofol group (61 ± 5 vs. 57 ± 9; SMD = 0.55, 95% CI: 0.17–0.93; *p* = 0.005). Additionally, the ciprofol group exhibited a significantly lower rate of perioperative vasopressor utilization and a decreased overall number of administrations when compared with the propofol group (*p* = 0.007). A striking reduction in the incidence of injection pain was observed in the ciprofol group (1.8% vs. 27.3%; ARR = 25.5, 95% CI: 13.2–37.7%; *p* < 0.001). No statistically significant distinctions were found between the two groups with respect to the duration until LoC or the time required for complete recovery.

**Table 2:** Outcome measures for ciprofol vs. propofol.

Variables	Ciprofol (*n* = 55)	Propofol (*n* = 55)	SMD/ARR (95% CI)	*p*-value
Incidence of hypotension, *n* (%)	20 (36.4)	36 (65.5)	29.1 (11.2–47.0)	0.002
Average MAP (mmHg)	80 (10)	77 (7)	0.35 (−0.03–0.72)	0.071
Minimum HR (beat min^−1^)	61 (5)	57 (9)	0.55 (0.17–0.93)	0.005
Count of cases needing any vasopressor, *n* (%)	18 (32.7)	32 (58.2)	25.5 (7.5–43.4)	0.007
One administration of vasopressor	6 (10.9)	13 (23.6)		
Two or more administrations of vasopressor	12 (21.8)	19 (34.6)		
Frequency of injection pain, *n* (%)	1 (1.8)	15 (27.3)	25.5 (13.2–37.7)	<0.001
Time to LoC (s)	57 (14)	53 (10)	0.33 (−0.05–0.70)	0.087
Time of complete recovery (min)	22 (6)	23 (7)	−0.15 (−0.53–0.22)	0.423

Data are presented as mean (SD), or number (%).

SMD, standardized mean difference; ARR, absolute risk reduction; CI, confidence interval; MAP, mean arterial pressure; HR, heart rate.

**Figure 2:**
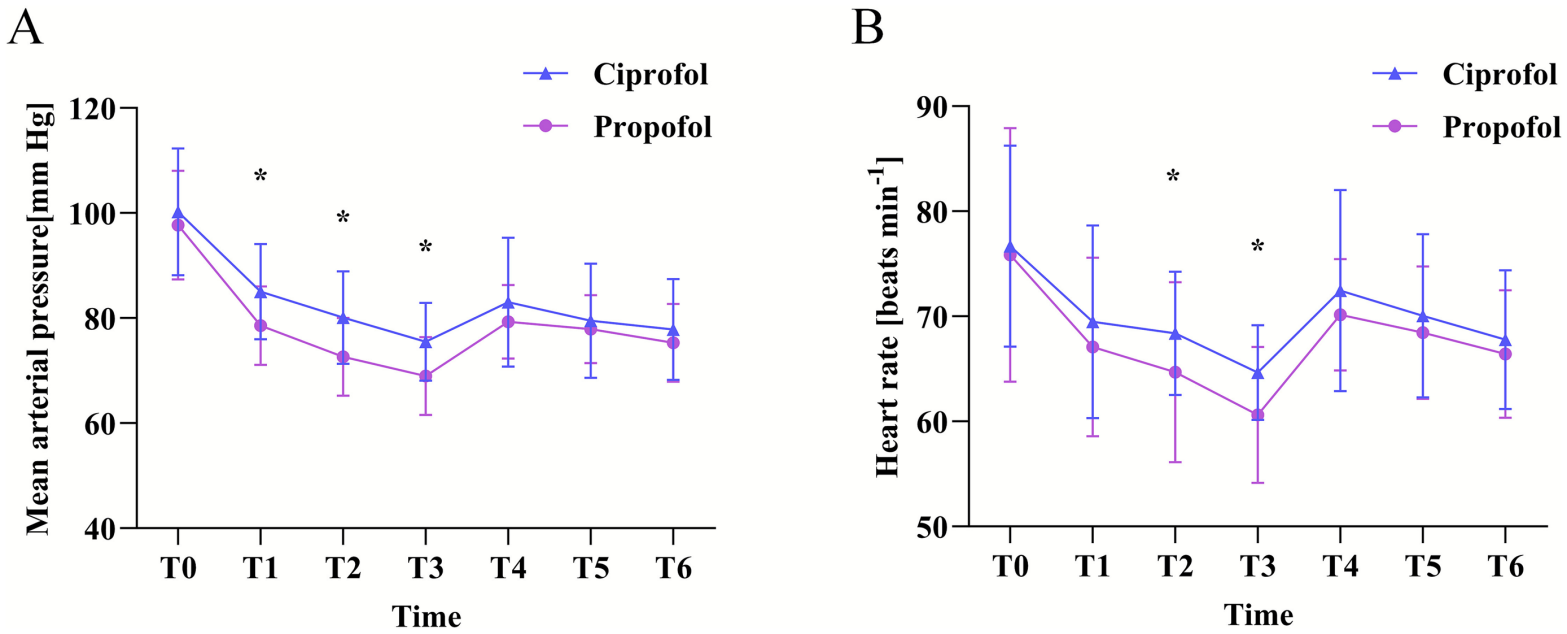
Changes in **(A)** the MAP and **(B)** the heart rate after induction of general anesthesia. All data are presented as mean with error bars showing the standard deviation. The baseline value is the mean of two measurements acquired immediately after the subjects entered the operating theater: pre-induction (T0), when BIS is ≤60 (T1), 1 min after induction (T2), intubation (T3), 3 min after intubation (T4), and 5 min after intubation (T5). Symbols “^*^”, “^**^”, and “^***^” indicate significance levels of *p* < 0.05, *p* < 0.01, and *p* < 0.001, respectively.

During the anesthesia induction period, the fluctuation amplitude of MAP in the ciprofol group was significantly smaller than that in the propofol group, indicating that ciprofol has advantages in maintaining hemodynamic stability. At the T1, T2, and T3 time points, the mean MAP values in the ciprofol group were significantly higher than those observed in the propofol group (*p* < 0.05). Additionally, HR measured at T2 and T3 was markedly higher in the ciprofol group by comparison (*p* < 0.05) (Figure 2).

**Figure 3:**
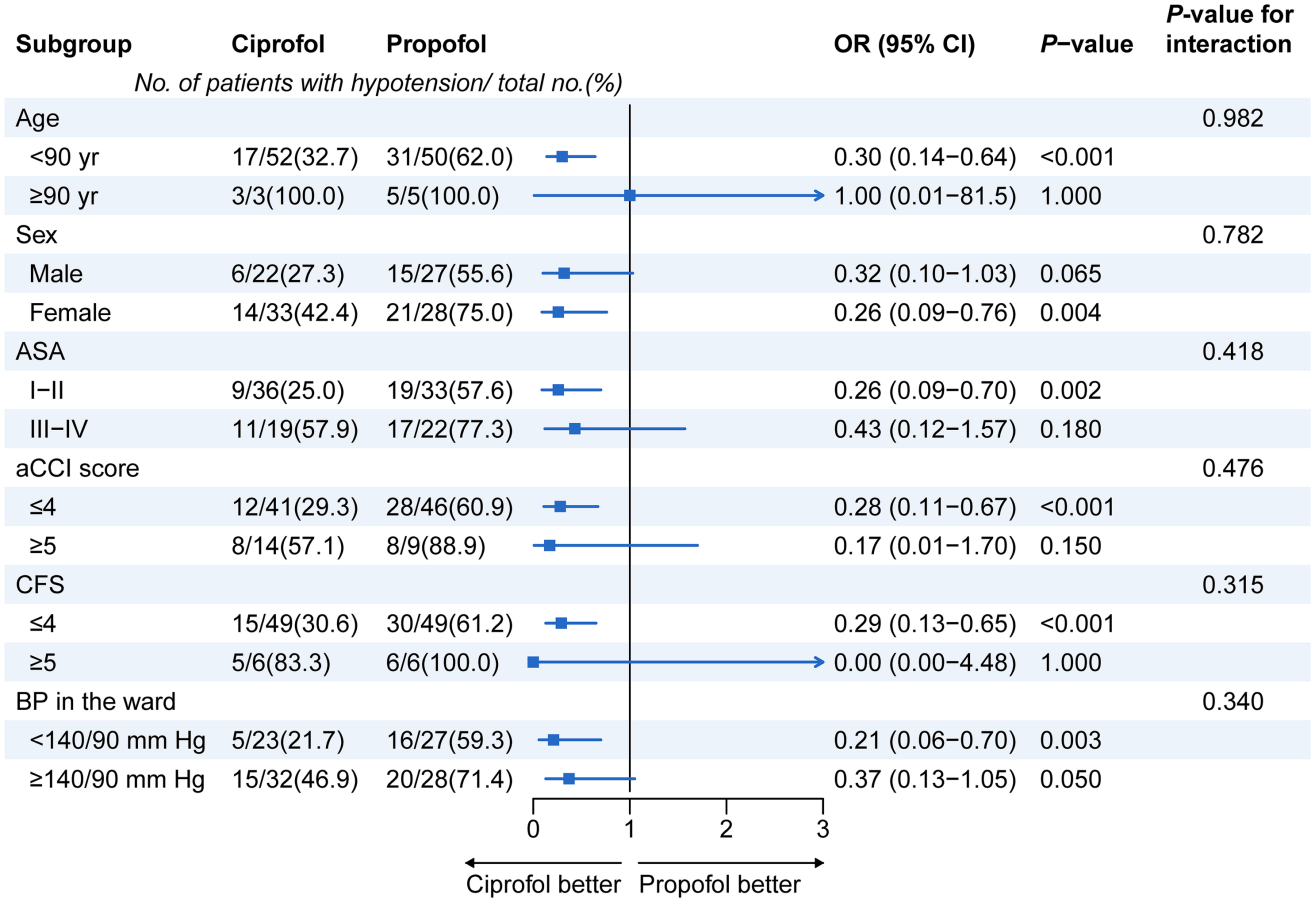
Subgroup analysis for the incidence of hypotension from the start of anesthetic administration until 5 min after tracheal intubation. BP, blood pressure; aCCI, age-adjusted Charlson Comorbidity Index; CFS, Clinical Frailty Scale; CI, confidence interval; OR, odds ratio.

Subgroup assessments were performed to explore the incidence of PIH in elderly patients receiving either ciprofol or propofol, with stratification based on age, gender, ASA classification, aCCI, CFS, and baseline ward blood pressure. As shown in the forest plot in Figure 3, across all pre-defined subgroups, the incidence of hypotension seen in the ciprofol group was markedly lower than in the propofol group. Statistical significance was noted in the following subgroups: patients aged under 90 years (OR = 0.30, 95% CI: 0.14–0.64; *p* = 0.001), those with an aCCI ≤4 (OR = 0.28, 95% CI: 0.11–0.67; *p* = 0.001), individuals with a CFS ≤4 (OR = 0.29, 95% CI: 0.13–0.65; *p* = 0.001), and patients with baseline BP below 140/90 mmHg (OR = 0.21, 95% CI: 0.06–0.70; *p* = 0.003). All interaction *p*-values in the subgroup analyses were >0.05, signifying that patient variables (age, sex, ASA class, aCCI, CFS, baseline BP) did not significantly alter the benefit of ciprofol over propofol in mitigating the risk of hypotension.

## 4 Discussion

This single-center retrospective study systematically assessed differences between the new intravenous anesthetic ciprofol and the conventional agent propofol in terms of hypotension incidence and hemodynamic stability following general anesthesia induction in elderly patients. The primary finding of the study showed that, compared with propofol, the application of ciprofol during general anesthesia induction in older individuals was linked to a significantly lower hypotension rate (36.4% vs. 65.5%; *p* = 0.002), corresponding to an ARR of 29.1%. In addition, ciprofol showed significant advantages in reducing injection pain, decreasing the need for vasoactive drugs, alleviating heart rate inhibition, and maintaining a more stable hemodynamic trajectory. These results provide strong evidence-based medical evidence for the superior safety of ciprofol in elderly anesthesia induction, especially in terms of hemodynamic stability.

Elderly patients have degenerated physiological functions, often have multiple organ dysfunctions, are sensitive to anesthetic drugs, and the incidence of PIH can be as high as 70% (21). This study observed a high incidence of PIH (65.5%) in the propofol group, confirming that the elderly population is highly susceptible to the circulatory inhibitory effect of propofol due to decreased physiological reserve, weakened vascular elasticity, impaired autonomic nerve regulation, and increased drug sensitivity. The significant reduction in the incidence of PIH due to ciprofol is closely related to its unique pharmacological properties. Ciprofol introduces a cyclopropyl group into the molecular skeleton of propofol to form a chiral center, which significantly enhances the affinity for GABAA receptors (16, 17). This structural modification greatly reduces the clinical dose required to achieve the same anesthetic depth (13). The core mechanism of propofol-induced hypotension lies in its direct dose-dependent myocardial contractility inhibition and peripheral vasodilation (9, 10). Therefore, ciprofol significantly reduces the effective anesthetic dose, thereby reducing the drug load on the cardiovascular system from the source, which is the fundamental reason why its PIH incidence is significantly lower than that of propofol. Findings from this study provide additional robust RCT data supporting previous observational studies by Ding et al. (16) and Liang et al. (17) on the safety and efficacy of ciprofol in elderly patients.

During the induction phase, the ciprofol group demonstrated a significantly higher mean MAP than the propofol group (*p* < 0.05), along with a decreased range of MAP fluctuations. Furthermore, the minimum heart rate recorded in the ciprofol group was markedly higher than that in the propofol group (61 ± 5 vs. 57 ± 9, *p* = 0.005). The mild circulatory inhibitory effect of ciprofol may be attributed to its strong efficacy and low required dosage (22). In the ciprofol group, the utilization rate and overall frequency of perioperative vasopressors were markedly lower than in the propofol group (*p* = 0.007), which is a direct clinical manifestation of its better hemodynamic stability. Reducing the use of vasoactive drugs also avoids the potential side effects of these drugs themselves, such as tachycardia and increased myocardial oxygen consumption caused by ephedrine or reflex bradycardia and splanchnic vasoconstriction caused by phenylephrine, which affect organ perfusion (23). This further enhances the safety of ciprofol induction.

Findings of this study showed that the incidence of injection-related pain associated with ciprofol was significantly lower than that linked to propofol. This observation aligns with research results reported by Qin et al. (24), Zhong et al. (25), and other scholars in the field. This phenomenon could be attributed to the addition of a cyclopropyl side chain to ciprofol’s structure compared to propofol (12). This asymmetrical molecular configuration enhances ciprofol’s lipid solubility, reduces the concentration of free drug in its aqueous phase, and the lower dosage of ciprofol administered further decreases the risk of injection-related pain. In contrast, propofol is primarily metabolized in the liver, with cytochrome P450 (CYP) 2B6 and glucuronyltransferase (UGT) serving as its key metabolic enzymes. After binding with glucuronic acid, it forms an inactive metabolite, which is mainly excreted through the kidneys via urine (26). Results also indicated no statistically significant difference between the two groups in terms of the time required to induce LoC or achieve full recovery. This result is consistent with earlier research (27). This similarity may stem from the comparable chemical structures and pharmacokinetic characteristics of ciprofol and propofol.

The highlight of this study design lies in its in-depth pre-specified subgroup analysis, which aims to explore whether the advantages of ciprofol are affected by specific patient characteristics. The analysis covers age, gender, ASA grade, aCCI score, CFS score, and baseline BP level. Across all pre-specified subgroups, the ciprofol group demonstrated a reduced risk of hypotension in comparison with the propofol group. This benefit attained statistical significance in several crucial subgroups, including those with age <90 years, aCCI ≤4, CFS ≤4, and baseline BP <140/90 mmHg. It is particularly critical that the interaction *p*-values of all subgroup analyses were >0.05, clearly indicating that the significant advantage of ciprofol over propofol in reducing the risk of hypotension during induction is not affected by these baseline demographic and clinical characteristics. This means that regardless of the age stratification, frailty degree, number of comorbidities, or baseline BP level of elderly patients, choosing ciprofol induction can more reliably obtain better hemodynamic protection.

This study has several limitations. First, it is a single-center retrospective study, which may be subject to selection bias. Meanwhile, as it is impossible to completely exclude subjective factors and unobserved covariates during clinical diagnosis and treatment, the study results may be affected by potential observer bias and confounding bias. In the future, it is necessary to expand the sample size through multicenter clinical trials to comprehensively verify the benefit-risk ratio of ciprofol in a broader population of elderly patients. Second, although blood pressure and heart rate were monitored, advanced hemodynamic assessment techniques [e.g., transesophageal echocardiography (TEE) or non-invasive cardiac output monitoring (NICOM)] were not employed. Consequently, the study cannot precisely delineate whether ciprofol’s blood pressure-stabilizing effect primarily stems from mitigating myocardial depression, reducing vasodilation, or a combination of both mechanisms. Upcoming studies that incorporate monitoring of cardiac output (CO) and systemic vascular resistance (SVR) will further clarify the circulation-stabilizing mechanisms of ciprofol. Third, this study mainly focuses on short-term outcome indicators such as hemodynamics during the induction period and PIH. Whether the clinical advantages of ciprofol in improving circulatory homeostasis can be further translated into a reduction in the incidence of severe postoperative complications (e.g., myocardial injury, acute kidney injury, and postoperative delirium), shortened length of hospital stay, and decreased mortality rate remains to be verified by long-term follow-up through large-scale, multicenter prospective randomized controlled trials in the future. Such prospective evidence will provide important support for the comprehensive evaluation of the organ-protective effects and long-term clinical benefits of ciprofol in elderly patients.

## 5 Conclusion

This study confirms that ciprofol has better hemodynamic stability than propofol during general anesthesia induction in THA elderly patients, especially in reducing the risk of hypotension, reducing the use of vasoactive drugs, and reducing injection pain, and this advantage is universal in elderly patients with different ages and comorbidities. This provides new evidence for the selection of anesthetic induction drugs for elderly orthopedic surgery patients.

## Data Availability

The original contributions presented in the study are included in the article/Supplementary material, further inquiries can be directed to the corresponding author.
