# Supplementary material for: Effect of ciprofol on the incidence of hypotension during induction of general anesthesia in elderly patients undergoing total hip arthroplasty: single-center retrospective study
Source: Front Med (Lausanne). 2026 Feb 9;13:1764580. doi: 10.3389/fmed.2026.1764580 (PMC12926384; doi:10.3389/fmed.2026.1764580)
Supplement: Supplementary file 1 [file Table_1.docx]

**Table S1: Modified Observer's Assessment of Alertness/Sedation (MOAA/S) Scale**

| Score | Responsiveness Level | Operational Definition |
| --- | --- | --- |
| 5 | Alert | Responds readily to name spoken in normal tone |
| 4 | Lethargic response | Responds to name spoken in normal tone with sluggish eye |
|  |  | opening/movement |
| 3 | Responds only to loud/repeated name | Requires loud voice and/or repeated name calling to elicit response |
| 2 | Responds only to mild prodding/shake | Responds only after mild prodding or shoulder shake |
| 1 | Responds only to painful stimulus | Responds only to trapezius squeeze or another noxious stimulus |
| 0 | Unresponsive | No response to trapezius squeeze |

**Table S2: Clinical Frailty Scale (CFS)**

| Score | Frailty Category | Clinical Descriptors |
| --- | --- | --- |
| 1 | Very fit | Elite fitness; regular vigorous exercise |
| 2 | Well | Active without disease limitations |
| 3 | Managing well | Comorbidities controlled without functional impairment |
| 4 | Vulnerable | Subjective fatigue; limited strenuous activities |
| 5 | Mildly frail | Needs assistance with IADLs (e.g., transportation, heavy chores) |
| 6 | Moderately frail | Needs assistance with ≥1 ADL (bathing, dressing) or mobility impairment requiring gait aid |
| 7 | Severely frail | Completely dependent for personal care (ADLs); housebound |
| 8 | Very severely frail | Completely dependent; approaching end-of-life (life expectancy <6 months) |
| 9 | Terminally ill | Life expectancy <6 months (not acutely reversible) |

**Table S3: Age-Adjusted Charlson Comorbidity Index (aCCI)**

**A. Comorbidity Weights**

| Condition | Weight | Diagnostic Criteria |
| --- | --- | --- |
| Myocardial infarction | 1 | History + ECG/Q-wave evidence |
| Congestive heart failure | 1 | NYHA Class II-IV or EF < 40% |
| Peripheral vascular disease | 1 | Claudication + ABI < 0.9, amputation, or revascularization |
| Cerebrovascular disease | 1 | Stroke with residual deficit or TIA |
| Dementia | 1 | MMSE < 24 or clinical diagnosis |
| Chronic pulmonary disease | 1 | FEV1 < 65% predicted, O₂ dependency, or pulmonary hypertension |
| Rheumatologic disease | 1 | RA, SLE, or vasculitis requiring immunosuppression |
| Peptic ulcer disease | 1 | Endoscopy-confirmed ulcer |
| Mild liver disease | 1 | Chronic hepatitis or cirrhosis without portal hypertension |
| Diabetes without complications | 1 | Diet/oral agent-controlled |
| Diabetes with complications | 2 | Nephropathy, retinopathy, neuropathy, or PVD |
| Hemiplegia | 2 | Persistent motor deficit ≥ 6 months |
| Moderate/severe renal disease | 2 | Creatinine > 3.0 mg/dL, dialysis, or transplant |
| Localized solid tumor | 2 | Non-metastatic malignancy active within 5 years |
| Leukemia | 2 | Acute or chronic (non-lymphocytic) |
| Lymphoma | 2 | Hodgkin’s or non-Hodgkin’s |
| Moderate/severe liver disease | 3 | Cirrhosis with portal hypertension or variceal bleeding |
| Metastatic solid tumor | 6 | Distant metastases |
| AIDS | 6 | HIV infection with CD4 < 200 or opportunistic infection |

**B. Age Adjustment**

| Age Group (years) | Additional Points |
| --- | --- |
| <50 | 0 |
| 50–59 | 1 |
| 60–69 | 2 |
| 70–79 | 3 |
| ≥80 | 4 |

**Total aCCI Score = A + B**
